# Do Physical Activity and Personality Matter for Hair Cortisol Concentration and Self-Reported Stress in Pregnancy? A Pilot Cross-Sectional Study

**DOI:** 10.3390/ijerph17218050

**Published:** 2020-11-01

**Authors:** Dagmara Budnik-Przybylska, Radosław Laskowski, Paulina Pawlicka, Paulina Anikiej-Wiczenbach, Ariadna Łada-Maśko, Anna Szumilewicz, Franciszek Makurat, Jacek Przybylski, Hideaki Soya, Maria Kaźmierczak

**Affiliations:** 1 Department of Sport Psychology, Institute of Psychology, Faculty of Social Sciences, University of Gdańsk, 80-309 Gdańsk, Poland; dagmara.budnik-przybylska@ug.edu.pl (D.B.-P.); franciszek.makurat@ug.edu.pl (F.M.); jacek.przybylski@ug.edu.pl (J.P.); 2 Department of Physiology and Biochemistry, Faculty of Physical Culture, Gdansk University of Physical Education and Sport; 80-336 Gdańsk, Poland; radoslaw.laskowski@awf.gda.pl; 3 Department of Cross-Cultural Psychology and Psychology of Gender, Institute of Psychology, Faculty of Social Sciences, University of Gdańsk, 80-309 Gdańsk, Poland; paulina.pawlicka@ug.edu.pl; 4 Psychological Counseling for Rare Genetic Diseases Institute of Psychology, Faculty of Social Sciences, University of Gdańsk, 80-309 Gdańsk, Poland; paulina.anikiej@ug.edu.pl; 5 Department of Developmental Psychology and Psychopathology, Institute of Psychology, Faculty of Social Sciences, University of Gdańsk, 80-309 Gdańsk, Poland; ariadna.lada@ug.edu.pl; 6 Department of Fitness, Faculty of Physical Culture, Gdansk University of Physical Education and Sport, 80-309 Gdańsk, Poland; anna.szumilewicz@awf.gda.pl; 7 Sports Neuroscience Division, Advanced Research Initiative for Human High Performance, Faculty of Health and Sport Sciences, University of Tsukuba, 305-8574 Tsukuba, Japan; soya.hideaki.gt@u.tsukuba.ac.jp; 8 Department of Family Studies and Quality of Life, Institute of Psychology, Faculty of Social Sciences, University of Gdańsk, 80-309 Gdańsk, Poland

**Keywords:** pregnancy, stress, hair cortisol concentration, physical exercise, personality

## Abstract

Background: Physical activity reduces psychosocial stress in pregnant women. Stress levels might be self-reported (psychosocial) or measured with biomarkers, one of which is hair cortisol concentration (HCC). Additionally, personality has been associated with stress and physical activity. Methods: The first aim of our study was to explore the differences in self-reported stress assessed by the Perceived Stress Scale (PSS) and in HCC with regard to physical activity level in pregnant (N *=* 29) and non-pregnant (N = 21) women. The second aim was to analyze the correlations among perceived stress, HCC, frequency of exercise and personality in the two groups separately. Results: There was a significant difference in frequency of exercise and self-reported stress between the two groups, with a lower level in pregnant women, but no differences in HCC and in personality were found. In the group of pregnant women, there was a significant negative correlation between HCC and frequency of exercise sessions, with the latter correlating positively with openness to experience. In the group of non-pregnant women, perceived stress negatively correlated with extraversion, agreeableness and emotional stability. HCC correlated negatively with conscientiousness. Conclusions: Our findings indicate the importance of physical activity programs dedicated to pregnant women for their life quality.

## 1. Introduction

Maternal prenatal psychological distress and lifestyle during pregnancy influence child development and may result in potentially permanent changes and lifelong consequences [1]. The majority of studies explore the role of self-reported stress during pregnancy. Prenatal psychosocial stress has been linked to reported lifestyle and health behaviors, but the physical activity of pregnant women has rarely been controlled [2]. Heightened distress has been quite consistently linked to impaired psychosocial and neurobiological development of a child [3,4]. The biological mechanism linking distress to child outcomes has been connected with the mediating effect of maternal cortisol concentration [5,6,7]. 

One of the biomarkers of chronic maternal stress response from the last month is the cortisol level in hair samples measured non-invasively. Temperature and other external variables like noise, as well as social interaction, do not influence such a measure of stress [8,9,10]. However, the results of prior studies concerning the association between hair cortisol concentration (HCC) and self-reported stress in pregnant women are often contradictory and might be influenced by the characteristics of samples or analysis protocols [11]. Some research on pregnant women indicated that hair cortisol was positively associated with perceived stress [12,13,14]. Still, for example, Bowers et al. [15] confirmed a positive association between self-reported stress and hair cortisol but only in the group of pregnant women who experienced high levels of childhood adversity. Thus, focusing on the specific characteristics of pregnant women and on a particular method of HCC analysis might facilitate understanding of associations between various assessments of stress in this unique time of life. 

In this paper, we concentrate on the role of physical activity in prenatal maternal stress. The impact of HCC on a growing fetus is not unitary and depends on various biological and psychosocial factors [2,11]. Studies have shown that physical exercise is one of the mechanisms substantially decreasing cortisol concentrations in various populations [16]. With regard to pregnant women, the results are inconsistent. For example, Garcia-Leon et al. [17] observed that HCC does not depend on the level of physical activity in pregnant women. In turn, Newham et al. [18] found that, despite the increase in cortisol levels as pregnancy developed, a single exercise session significantly lowered the level of this hormone in saliva. Physical activity in pregnancy additionally has been shown to enhance serotonin production and increase endorphin levels [19,20], and aerobic exercises help maintain mental and physical well-being in pregnancy [21,22,23]. 

The effectiveness of exercise is related to its components: frequency, intensity, time, type and progression [24]. In experimental studies, it was observed that exercise frequency may substantially differentiate the physiological response to an exercise program in pregnant women [25]. It can be assumed that the mechanism of lowering cortisol levels by regular prenatal exercise will also depend on its frequency. Based on a systematic review, Bessera et al. [26] concluded that exercise frequency was one of the strong factors influencing the reduction in cortisol levels in individuals with major depressive disorders. So far, there is no data on this issue from pregnant women. 

Personality traits may contribute to physical activity and responses to stress. There is evidence that lower neuroticism (higher emotional stability), higher conscientiousness, extraversion and openness to experience, as factors from the Five Factor Model of Personality, are associated with more frequent physical activity [27]. In turn, a sedentary lifestyle is associated with an increased risk of poor physical and mental health. Higher neuroticism and lower conscientiousness corelate with more time spent in sedentary behaviors. More neurotic individuals tend to set more avoidance-related goals and more frequently experience negative emotions and distress. Individuals scoring high in conscientiousness are better organized, disciplined and internally motivated for physical activity than those scoring low. Additionally, extraverted people enjoy positive emotions and more frequently engage in physical activity. Similarly, those who are more open to experience engage in a wide variety of activities and are motivated by health and fitness goals [27]. Moreover, a positive correlation between neuroticism and psychological stress has been reported, and a combination of low levels of neuroticism with high levels of conscientiousness has represented an advantageous profile for coping with stress [28,29].

Therefore, in the reported cross-sectional pilot study, we aimed at examining self-reported stress and HCC in pregnant women, as compared with the comparative group, and their associations with the frequency of exercise. Secondly, we also compared the two samples in terms of frequency of exercise. Due to the contradictory results of earlier studies, we did not hypothesize, but we explored whether there was a positive association between self-reported stress and HCC in both groups. We expected negative associations between both self-reported stress and HCC and the frequency of exercise undertaken by the studied women. We also analyzed participants’ general personality factors from the Five Factor Model as possible correlates of both stress and physical activity [27,28,29]. We expected that emotional stability, higher conscientiousness, openness to experience and extraversion would positively correlate with exercise frequency in both samples. Higher emotional stability and conscientiousness should correlate with lower stress, regardless of the measurement.

## 2. Material and Methods

### 2.1. Participants

Fifty women, group 1 consisting of 29 pregnant and group 2 consisting of 21 non-pregnant women, took part in the study. All participating women were of reproductive age (non-pregnant: M_age_ = 25; SD = 2; pregnant: M_age_ = 30; SD = 4; Z= −4.33, *p* < 0.001), and the average age in group 1 was higher (as the postponement of childbirth until older age has been observed in Poland [30]). Taking into account the length of reproductive age (from 15 to 49 years [31]), the observed age difference between the groups can be regarded as of no clinical significance. 

The inclusion criteria for both samples were:

- declared general good health (none of the women reported exercise contraindications)
- no addictions to tobacco, alcohol, drugs or psychoactive substances
- no chronic diseases (including gestational diabetes mellitus or hypertension) and no reported pharmacological treatment, such as glucocorticosteroids or selective serotonin reuptake inhibitors.

There was an additional inclusion criterion for pregnant women:

- the second or third trimester of pregnancy with a first child (single pregnancy).

In this study, only those women who agreed to give hair samples were included. 

Participants who did not meet any of the inclusion criteria were excluded from the study.

### 2.2. Measurement

#### 2.2.1. Self-reported Psychosocial Stress

The Perceived Stress Scale (PSS)-10 [32,33] was used to measure stress experienced in the last month. It contains 10 questions about different subjective feelings related to personal problems and events, behavior and ways of dealing with them. The participant responds to ten items on a 5-point Likert scale (0 = never, 1 = almost never, 2 = sometimes, 3 = fairly often, and 4 = very often). Six of the items are negatively worded (e.g., “How often have you been upset because of something that happened unexpectedly?”), and four are positively worded (e.g., “How often have you felt that you were on top of things?”). A total PSS-10 score is the sum of all 10 items, with reverse scoring of the four positively worded items. A higher total score indicates a higher level of perceived stress. 

Cronbach’s alpha coefficient for the Polish PSS-10 = 0.89.

#### 2.2.2. Hair Cortisol Concentration (HCC)

Small hair strands were taken from the scalp near the posterior vertex region of each participant. Cortisol was assessed separately for each of the 1-cm segments. Based on an approximate hair growth rate of 1 cm per month [34], a 1-cm hair segment should represent a period of about one month. The procedure of hair segment analysis followed a modified version of the laboratory protocol previously described in [35]. Each hair segment was washed twice with 4 mL isopropanol for 3 min to remove external contaminants from the outer hair. The washed hair was then placed in a tissue paper and dried for 12 h. Next, the hair was powdered in liquid nitrogen, and the powder was poured into the samples. Then we heated it at 50 °C for 2 h. For steroid extraction, 1 ml of methanol was added; then we heated it at 50 °C for 16 h. Following steroid extraction, the samples were spun in a microcentrifuge (7000 rpm for 30 s), and 1 mL of the clear supernatant was transferred into a new vial. The methanol was evaporated under a constant stream of nitrogen at 55 °C until the samples were completely dried. Finally, 0.4 mL phosphate buffer was added, and the vials were vortexed for 15 s. We determined cortisol using the DetectX, Cortisol Enzyme immunoassay kit (Arbor Assays, MI, 48108-3284 USA) and the ELISA method [36].

#### 2.2.3. Personality

The Ten-Item Personality Inventory in Polish adaptation (TIPI [37]; TIPI-PL [38]), a short method built of 10 items, was used to measure the Five Factors of Personality: extraversion, agreeableness, conscientiousness, emotional stability and openness to experience. A participant responds to ten items referring to the statement: “I perceive myself as a person...”, giving her answer according to a 7-point scale, where 1 means “I definitely disagree”, 2 “I rather disagree”, 3 “I disagree slightly”, 4 “I neither agree nor disagree”, 5 “I agree slightly”, 6 “I rather agree” and 7 “I strongly agree”. Example items include, “I see myself as extraverted, enthusiastic” (extraversion) and “I see myself as dependable, self-disciplined” (conscientiousness). Five items are recoded. Due to its short form, the scale is increasingly being used for research purposes. It should be emphasized that TIPI (original, as well as the Polish adaptation) measures general personality factors with only two items for each factor, one positive and one negative, which decreases the Cronbach’s alpha values. Cronbach’s alphas of all subscales are similar to those of the original version [37,38]: Cronbach’s alpha = 0.37 for openness to experience, Cronbach’s alpha = 0.39 for agreeableness, Cronbach’s alpha = 0.65 for emotional stability, Cronbach’s alpha = 0.70 for conscientiousness and Cronbach’s alpha = 0.59 for extraversion.

#### 2.2.4. Frequency of Exercise Sessions

Women also reported their frequency of exercise, expressed in the number of exercise sessions usually performed per week. We defined “exercise” as a “form of physical activity that is planned, structured, repetitive, and performed with the goal of improving health or fitness” [39] (p. 18). As an “exercise session”, we considered each time a woman undertook exercise. The frequency of exercise was the sole exercise component analyzed in the study. Firstly, it is related to the changes in cortisol levels [26]; secondly, it is the easiest for exercise participants to properly report and therefore less prone to bias in a non-interventional study. Other components of an exercise program, especially exercise intensity, are difficult to self-assess by inexperienced study participants. 

### 2.3. Procedure

All the women participated in the larger research project entitled the HEART research project (Hormonally mediated Empathy role for Affiliative Response towards infant Tears). 

The women were contacted via antenatal schools in the Pomeranian area of Poland and via social media. All volunteers filled in the recruitment form to answer questions concerning the inclusion criteria. Subsequently, all participants who met the inclusion criteria were invited to take part in the study. Fifty-five women agreed to give their hair samples, of which five were excluded during hair segment analysis.

The study was conducted in the laboratory situated in the Institute of Psychology at the University of Gdańsk and performed according to the principles of the Helsinki Declaration. Personal information has been processed in accordance with the Polish Personal Information Protection Act, based on European legislation. The participants signed the informed consent before testing. The research protocol for the HEART project received ethical approval from the Independent Bioethics Committee for Scientific Research at the Medical University of Gdańsk, Poland (permission #NKBBN/154/2017) and the Ethics Committee at the Institute of Psychology, University of Gdańsk, Poland (permission #4/2016). Additionally, for the inclusion of the HCC measurement, a separate ethical approval was received from the Ethics Committee at the Institute of Psychology at the University of Gdańsk, Poland (permission #8/2017).

### 2.4. Statistical Analysis 

The Shapiro-Wilk test was applied to assess the homogeneity of dispersion from the normal distribution. The variables did not present normal distribution and were described by median (Me), minimum (min.) and maximum (max.) values. The data analysis was carried out using IBM SPSS Statistics 26 for a Mann-Whitney U test in testing the differences between the two analyzed groups and Spearman Rho correlations among perceived stress, HCC, frequency of exercise and personality in pregnant and non-pregnant women. The significance level was set at *p* ≤ 0.05.

## 3. Results

According to the first aim of the study (examining self-reported stress and HCC in pregnant women, as compared with the non-pregnant group, and their associations with exercise frequency), the Mann-Whitney U test indicated that there were significant differences only in perceived stress (*U*= 155.00, *Z*= −2.82, *p* = 0.005), with a lower level found in pregnant as compared with non-pregnant women. The HCC did not differentiate the groups. 

The differences between the group of pregnant and the group of non-pregnant women are presented in Table 1.

Next, we observed a significant negative correlation between HCC and exercise frequency in the group of pregnant women (*rho* = −0.488, *p* = 0.003), whereas there was no such correlation in the group of non-pregnant women. On the other hand, self-reported stress did not correlate with exercise frequency in both groups. Pregnant women were substantially more often active than the non-pregnant women (see Table 1). 

The analysis of the general personality factors of participants as possible correlates of bot stress and exercise frequency in the group of pregnant women indicated no associations between personality, self-reported stress and HCC. Only openness to experience correlated positively with exercise frequency (*rho* = 0.52, *p* = 0.002). 

In the group of non-pregnant women, three personality dimensions, extraversion, agreeableness and emotional stability, correlated negatively with perceived stress (*rho* = −0.449, *p* = 0.041 for extraversion, *rho* = −0.52, *p* = 0.016 for agreeableness and *rho* = −0.47, *p* = 0.032 for emotional stability). Conscientiousness correlated negatively with HCC (*rho* = −0.59, *p* = 0.005). There were no correlations between personality and exercise frequency.

The above results are presented in Table 2. 

## 4. Discussion

To the best of our knowledge, this is the first study that analyzed both self-reported psychosocial and physiological stress in pregnant, as compared with non-pregnant, women in the context of physical activity patterns and personality. 

The pregnant and non-pregnant groups did not differ with regard to hair cortisol concentration (HCC). However, self-reported stress assessed by the Perceived Stress Scale (PSS) was lower in pregnant women. There was also no significant relationship between HCC and the PSS score in both groups. This result contradicts the findings of previous studies in which hair cortisol positively correlated with perceived stress [13,14]. 

Interestingly, we discovered that the pregnant women who participated in the study exercised more often than the non-pregnant women. Currently, prenatal physical activity is considered a necessary condition for the proper course of pregnancy and child development, as well as the prevention of perinatal complications [40,41]. Therefore, due to the pervasively emphasized value of physical activity, the self-reported psychosocial stress in a more active group of pregnant women might have been lower. 

Moreover, in pregnant women (but not in the non-pregnant ones), xercise frequency correlated negatively with HCC. This outcome corresponds with the already mentioned findings indicating that, regardless of the stage of pregnancy, a single session of exercise has an immediate effect on lowering salivary cortisol levels in pregnant women [18]. Some authors have emphasized that prevention and intervention programs aimed at lowering distress in pregnancy and/or enhancing self-regulation are much needed [2,42] and might involve physical activity. Based on our results, we may conclude that much attention should be paid to the frequency of exercise sessions performed by women in such programs. The effects of physical exercise on self-reported stress in both samples and on HCC in non-pregnant women remain to be confirmed in larger samples. It can be assumed that the exercise stimuli were too weak to decrease the production of cortisol in the non-pregnant group or self-reported stress in both samples. 

Another aspect of the analyses concerned general personality factors as possible correlates of both stress (self-reported and HCC) and physical activity. The results indicated that the number of exercise sessions per week correlated positively with openness to experience, which confirmed its associations with a greater need for stimuli and higher engagement with various types of activities, including physical exercise [27]. This relationship was, however, observed only in the group of pregnant women. Such a result may be connected with the specificity of the group of women who decided to participate in the study. 

Similarly, personality correlated with both self-reported stress and HCC only in the group of non-pregnant women. There is evidence that pregnancy strongly defines women’s behaviour. Sjögren, Widström, Edman and Uvnaus-Moberg [43] revealed significant changes during first pregnancy and lactation towards more relaxation and tolerance of monotony. Adversely, plenty of research indicated negative changes in behavior and mood during pregnancy [44]; therefore subjectively measured stress may not be a sufficient indicator of emotional state in pregnancy. 

HCC in the group of non-pregnant women was connected solely with a lower level of conscientiousness, which confirmed the results of selected previous studies (some studies indicated a lack of effects [45]). High conscientiousness is connected with high efficiency, good organization and self-discipline, which are socially promoted [46]. A lack of these might be linked to heightened physiological stress, yet this was subjectively not noticeable in our study. Additionally, in non-pregnant women self-reported psychosocial stress correlated with lower levels of extraversion, agreeableness and emotional stability. The obtained results may indicate that women who were more reserved, less outgoing, simultaneously competing with others and presenting higher levels of anxiety, anger or frustration perceived more psychosocial stress [47].

This study is not free from limitations. As it was a pilot study, the study group was small. However, obtaining agreement to a hair sample from women proved to be challenging, and a high frequency of women having their hair dyed, which was an exclusion criterion, posed an additional hindrance. Therefore, we conducted only simple statistical analyses. The specified level of physical activity was not an enrollment criterion, and, unexpectedly, the comparative group of non-pregnant women turned out to be less physically active than the pregnant group. Our research aim was not to examine factors influencing the differentiation of physical activity levels in pregnant and non-pregnant women. Therefore, we did not ask the participants either questions about their motives for undertaking the activity or the time to start the activity (e.g., whether the pregnant women had only started exercising after conception). Nevertheless, we obtained an interesting result. The higher frequency of exercise in pregnant women may reflect the positive changes in physical activity patterns after conception, resulting from the worldwide promotion of prenatal exercise in recent years [40]. The significant difference in the frequency of exercise leads to some hypotheses. For example, this study outcome may be related to the current obligation of obstetric care providers to encourage pregnant women to exercise [41,48]. In addition, exercise sessions are most commonly offered to women in antenatal education programs. However, in this study, it made it difficult to determine to what extent the changes in stress levels due to exercise differ between pregnant and non-pregnant women. In future studies, this research question is worth exploring in pregnant and non-pregnant women with the same level of exercise frequency. Additionally, a comparison of changes in hair cortisol concentrations and other stress parameters between groups of physically active and inactive pregnant women would pose an interesting research aspect. Self-reported exercise frequency was another weak point of our study. It would be valuable to conduct experimental research in which all components of an exercise program are measured by objective methods. 

Nevertheless, our results bring new knowledge on the subjective (self-reported) and objective (HCC) levels of stress in pregnant and non-pregnant women in relation to their physical activity patterns and personality. In particular, the apparent relationship between exercise frequency and reduced cortisol levels in pregnant women may have practical implications. This result should be included in the programming of exercises during the week in anticipation of the health effects in pregnant women particularly exposed to stress.

## 5. Conclusions

Physical activity does matter for hair cortisol concentration (HCC) but not for self-reported stress in pregnancy. Although the pregnant women declared significantly lower levels of stress as compared with non- pregnant women, the groups did not differ in HCC. Still, in pregnant women, the higher the frequency of exercising, the lower the HCC. The expectant women who were more open to experience were also more physically active. Referring to public health, the obtained results indicated the importance of programs of physical activity dedicated to pregnant women for their quality of life. 

## Figures and Tables

**Table 1:** Differences between the studied groups.

Variable	Pregnant Women Me (min-max)	Non-pregnant Women Me (min-max)	*p*
Hair Cortisol Concentration (pg/mg)	8.58 (1.70–70.52)	9.54 (2.28–55.17)	0.461
PSS	13.50 (6.00–22.0)	20.00 (9.00–35.00)	0.005
Frequency of exercise (number of exercise sessions per week)	2 (0–3)	0 (0–3)	0.014
Extraversion	6.00 (2.00–7.00)	5.50 (2.00–7.00)	0.766
Agreeableness	6.00 (3.50–7.00)	5.50 (3.00–6.50)	0.191
Conscientiousness	6.00 (2.50–7.00)	6.0 (2.50–7.00)	1.00
Emotional Stability	5.00 (1.50–6.50)	4.50 (1.50–6.50)	0.242
Openness to experience	4.50 (2.50–7.00)	4.50 (3.50–6.50)	6.60

Note: Me = median; min = minimum; max = maximum; *p* ≤ 0.05 was considered statistically significant.

**Table 2:** Correlations among perceived stress, hair cortisol concentration (HCC), frequency of exercise and personality in pregnant and non-pregnant women.

Group	Variable	PSS	Hair Cortisol Concentration (pg/mg)	Frequency of Exercise (Number of Exercise Sessions per Week)
Pregnant women	Extraversion	−0.21	−0.20	0.14
	Agreeableness	−0.35	−0.07	−0.09
	Conscientiousness	−0.22	0.05	−0.09
	Emotional Stability	−0.26	0.11	−0.26
	Openness to experience	−0.06	−0.32	0.539 **
	PSS	1.00	−0.04	0.11
	Hair Cortisol Concentration	−0.04	1.00	−0.591 **
	Frequency of exercise (number of exercise sessions per week)	0.11	−0.591 **	1.00
Non-pregnant women	Extraversion	−0.449 *	−0.21	0.04
	Agreeableness	−0.520 *	−0.11	−0.14
	Conscientiousness	−0.14	−0.590 **	0.25
	Emotional Stability	−0.469 *	−0.24	−0.04
	Openness to experience	0.07	0.25	−0.18
	PSS	1.00	−0.12	−0.07
	Hair Cortisol Concentration	−0.12	1.00	−0.34
	Frequency of exercise (number of exercise sessions per week)	−0.07	−0.34	1.00

Note: *p < 0.05; **p < 0.001.
